# Has the science of mindfulness lost its mind?

**DOI:** 10.1192/pb.bp.116.053686

**Published:** 2016-12

**Authors:** Miguel Farias, Catherine Wikholm

**Affiliations:** 1 Coventry University, Coventry, UK; 2 National Health Service, UK

## Abstract

The excitement about the application of mindfulness meditation in mental health settings has led to the proliferation of a literature pervaded by a lack of conceptual and methodological self-criticism. In this article we raise two major concerns. First, we consider the range of individual differences within the experience of meditation; although some people may benefit from its practice, others will not be affected in any substantive way, and a number of individuals may suffer moderate to serious adverse effects. Second, we address the insufficient or inconclusive evidence for its benefits, particularly when mindfulness-based interventions are compared with other activities or treatments. We end with suggestions on how to improve the quality of research into mindfulness interventions and outline key issues for clinicians considering referring patients for these interventions.

‘I therefore recommend meditation, just as I recommend the use of Jacobsen's relaxation method or other focusing and relaxing techniques, as a palliative, distraction method, and advise most of my clients to use it with discretion and not take it too seriously or view it as a general therapeutic method.’ Albert Ellis,^1^ p. 673.

Something has gone wrong with the science of mindfulness. The literature on its supposed mental and physical benefits is conceptually and methodologically precarious and has been divulged in a sensationalist way. Academic articles describe weak results as ‘encouraging’ and ‘exciting’; popular best-selling books about mindfulness, many of which are written by researchers, are bursting with magical promises of peace, happiness and well-being. The replacement of orange-robed gurus by white-collared academics who speak of the benefits of ‘being in the present moment’ is a powerful social phenomenon, which is probably rooted in our culture's desire for quick fixes and its attraction to spiritual ideas divested of supernatural elements. There is a misrepresentation of the place and value of meditation in the Buddhist tradition, including its depiction as a purely rational method of self-exploration,^2^ which would feel alien to countless past generations of Buddhists.^3^

There are two major types of problems with the attempts to study mindfulness. First, its scientific literature is plagued by conceptual and methodological shortcomings and the turning of a blind eye to the fact that individuals react differently to this technique. Second, we also have concerns about how it is being utilised by individuals with little formal training in mental health, and its branding (often against the alarming background of a global increase in mental illness) as the technique of choice to develop ‘mental fitness’. Our aim is not to engage in a damning critique of mindfulness, but simply to urge caution about its widespread use as a therapeutic technique, including its limitations, the lack of clear evidence about its benefits, and its ‘assembly-line’ approach based on a reductive understanding of the human mind.

## The thorn of individual differences

When the practice of meditation exploded in the West and was taken into the lab in the 1970s, the idea behind its efficacy was couched in a language of altered states of consciousness: meditating allowed an individual to enter a particular state of consciousness,^4^ which was associated with a range of physiological alterations and mental health benefits.^5^ Although the notion of ‘altered states of consciousness’ is no longer popular in the medical and psychological sciences, the supposed efficacy of mindfulness is rooted in a particular state of consciousness: a non-judgemental awareness of the stream of our experiences. There is an acknowledgement in the literature that individuals will vary in their dispositional or trait levels of mindfulness^6^ – in other words, how naturally gifted one is in achieving this state of consciousness – but nevertheless the underlying stance is a universalist one: the practice of mindfulness is regarded as an innate human cognitive ability which, when regularly engaged in, is beneficial to all. Given this universalist framework, it is perhaps not surprising that mindfulness researchers have generally turned a blind eye to the fact that individuals react differently to meditation techniques – and that these reactions may not always be positive.

Let us start by focusing on the benefits of mindfulness as a preventive treatment for recurrent depression, currently the only mental illness for which the National Institute for Health and Care Excellence (NICE) recommends the use of a mindfulness intervention. Mindfulness-based cognitive therapy (MBCT) was developed with the intent of treating individuals at risk for recurrent depression. The early studies showed that, when compared with a treatment-as-usual (TAU) group, mindfulness led to lower relapse rates for those with three or more episodes of depression.^7,8^ However, it increased the likelihood of relapse in individuals with two or fewer depressive episodes. But there is more. The last two major trials found that even patients with more than three episodes of depression react differently to it. Those who benefit the most were shown to be individuals with a personal history of childhood trauma and abuse, in other words, those most psychologically vulnerable.^9,10^ It is unclear what the reasons are for mindfulness being particularly effective within this subgroup of individuals with a high probability of depression relapse, but it certainly calls for a more nuanced recommendation by NICE.

## Potentially adverse effects of mindfulness

We have recently reviewed some of the evidence for what we call the ‘dark side of meditation’, which includes evidence of somatic, psychological and neurological problems associated with meditation practice.^11^ This is a surprisingly under-researched area, mostly consisting of case studies, but not exclusively. A cross-sectional study on the effects of intensive and long-term meditation reported that over 60% of individuals had at least one negative effect, which varied from increased anxiety to depression and full-blown psychosis.^12^ Qualitative research on mindfulness meditation shows that it may increase the awareness of difficult feelings and exacerbate psychological problems.^13^ One individual reported being suddenly confronted with material relating to a forgotten childhood trauma during his mindfulness practice:

‘I saw the depth of the pain that is buried things that have happened to me that have not been dealt with properly. It can be very scary to know there's that very strong thing in there’^13^ p. 853.

It can be argued that the emergence of difficult emotional material from mindfulness practice may be a positive, rather than an adverse circumstance. This will, of course, depend on the context in which these feelings and memories emerge – if it happens in a therapeutic context, it may very well be; but if the person is alone or doing mindfulness in a group setting without a trained mental health clinician, a positive outcome is more unlikely and it may simply result in unexpected distress.

Why do some people react badly to meditation? A possible explanation is that it amplifies inner problems; if one has a ‘disposition’ to depression, bipolar disorder or psychosis, meditation may heighten it. This amplification thesis, however, is purely speculative and based on a biased positive understanding of mindfulness. Another explanation is that mindfulness is not only about ‘being aware’ but may also challenge the ordinary sense of self. We call this the ego-rattling hypothesis. Meditation techniques, including mindfulness, were originally developed to assist with bringing about a deep change in how individuals perceive themselves, others and the surrounding world. It is then not entirely surprising that a person might experience emotional difficulties as a result. For example, it has been found that after an 8-week mindfulness-based stress reduction (MBSR) course, some participants experienced increased stress and depression.^14^ One experimental study, which used the Trier Social Stress Test, found that a short mindfulness intervention with healthy individuals led to increased biological stress when compared with an active control group.^15^

Individual differences in mindfulness, including the potential for adverse effects, should not be regarded as the elephant in the room. Their study is crucial if we are to advance our knowledge of the real therapeutic potential of mindfulness. We must understand for whom and under what circumstances it works and when it may be contraindicated. The neglect of individual differences has other obvious drawbacks: it weakens our conceptual understanding of mindfulness and severely limits the scientific usefulness of the plethora of studies that are searching for its benefits.

## The enthusiasm is ahead of the evidence

Contrary to popular opinion, the evidence for even the most ‘well-founded’ benefits of mindfulness is not consistent or conclusive. A recent comprehensive meta-analysis^16^ of randomised clinical trials showed that mindfulness interventions only led to moderate improvements in depression, anxiety and pain, and very small improvements in stress reduction and quality of life. There was no evidence that mindfulness had an effect on other variables, such as positive mood, attention, sleep or substance use. Further, when mindfulness was compared with other interventions, such as physical exercise or relaxation, it was not more effective. This confirms the result of an earlier meta-analysis,^17^ which found that mindfulness-based interventions did not lead to medium- or long-term (3 weeks to 3 years post-intervention) better clinical outcomes compared with relaxation or psychoeducation.

The enthusiasm surrounding mindfulness easily leads to reporting the evidence in a different way. Let us again take the case of mindfulness in the treatment of recurrent depression. Its last major trial was published with great media fanfare: ‘mindfulness is as effective as drugs for treating depression’ reported the *Daily Mail* (21 April 2015).^18^ What the media did not pick up on was that the study had in fact failed, as its hypothesis was that MBCT would be superior to antidepressant medication in preventing depression relapse.

The media also completely ignored the results of the previous major trial on MBCT for recurrent depression, a methodologically more sophisticated study and one with surprising results. Williams and colleagues^9^ employed a dismantling design to investigate the ‘active ingredient’ of MBCT by comparing a typical MBCT intervention with psychoeducation (similar in all aspects to MBCT, except it did not involve meditation) and a TAU group. The psychoeducation groups met for the same amount of time and learnt to recognise the warning signs of depression and disengage from them, exactly as in the MBCT group. All participants were assessed at 6, 9 and 12 months. The results: participants in the MBCT group were as likely to relapse as those in the psychoeducation and the TAU groups. The only participants for whom MBCT proved more effective were those with a higher frequency of childhood trauma and abuse.

There are many other findings in the literature that raise doubts about the long-term benefits of meditation. Two meta-analyses disconfirmed the expectation that continuous practice would lead to cumulative changes, both in emotional-cognitive domains^19^ and in brain structure.^20^ There are no obvious interpretations for the finding that the expected positive changes of mindfulness plateau after only some weeks of practice, rather than increase with time. But the contrary is also true – there is no clear rationale for why continuous mindfulness practice would keep improving well-being or cognitive abilities. A sort of magical rationale for the ‘power of mindfulness’ appears to be the underlying explanation. Continuous practice is supposed to add up in a mathematical way, making you more mindful, super aware, super controlled, super happy and eventually liberated from the illusion of the individual self. It is against the background of this expectation that researchers show surprise about the lack of a linear evolution of the benefits of mindfulness.

This grandiose expectation regarding the optimisation of human functioning through a meditation technique may be looked on as naive; but it is also dangerous. It is driving an enthusiasm to show the effects of mindfulness that runs way ahead of the modest evidence, as well as tainting our perception of the data. It does something else that we find worrying: it encourages a simplistic portrayal of the human mind and of our inner lives. A number of analogies are used to make mindfulness amenable to our modern mindset. A particularly popular one is to think of mindfulness as a ‘mind gym’: ‘Just as brushing your teeth or going for a run are well known ways of protecting general physical health, mindfulness exercises develop mental fitness and resilience’.^21^ It is unclear what these metaphors refer to – what exactly is mental fitness and how can mindfulness promote it? Is it a process of self-regulation mediated by improvements in attention and awareness? Or is it a process of reappraisal of one's thoughts and sense of self as unimportant or illusory?

## Recommendations and considerations

To improve the quality of research into mindfulness we first need clear and comprehensive theories of how it works that acknowledge the range of experiences people can have when they meditate. Second, regarding methodology, studies should involve active control groups, control for expectations, and seek to explore individual differences in more depth.

Until we have better-designed studies and evidence which can shed light on these areas, it is imperative that we consider mindfulness not as the ‘go-to’ approach for patients struggling with stress or recurrent depression, but as one possible therapeutic approach among others. It is important that we also speak openly about the potential for adverse effects in order to de-stigmatise the issue; surely the last thing we want is for a patient to feel that they ‘failed’ at using a technique, when the reality is that it worked differently for them than for another – and as yet, we do not know why.

Currently, there is no professional or statutory registration required to teach mindfulness-based interventions such as MBSR and MBCT, and no regulatory body which oversees the training of mindfulness teachers. The current popularity of mindfulness is encouraging the rushed, unregulated formation of thousands of teachers. Organisations offering training may set their ‘minimum requirements’ for those wanting to train, but these vary from organisation to organisation. Unlike other mainstream psychological interventions available in the UK in the National Health Service (such as cognitive-behavioural or systemic family therapy), you do not need to be a therapist or have any formal training in mental health to deliver mindfulness courses. In other words, some mindfulness teachers may be merely equipped to deliver a mindfulness package, in a group setting, and may have limited experience and expertise in identifying and managing mental health difficulties. Yet, given that mindfulness is promoted as a way to improve mental health, it is very likely that for individuals attending mindfulness groups in the community (or at school, or at work) many will be experiencing some level of mental health difficulties. For individuals experiencing common difficulties such as stress, anxiety or depression and considering paying for therapy or attending a mindfulness group, the combination of media hype and the comparative affordability of a mindfulness group may easily sway them to opt for this, potentially placing their mental health in the hands of someone who may lack adequate training and experience of working with psychological difficulties.

Key considerations for clinicians contemplating referring patients to mindfulness interventions include past experiences of meditative techniques, providing information as to the range of effects that may occur, ensuring that the individual has support in place to help them to manage difficult experiences should they occur, and giving them a choice as to whether this, or some other form of therapy, would be best suited to them.

Mindfulness has its place in therapy, as one of many techniques available to a trained clinician. However, we need to understand who it benefits and when, its merits and limitations. And we need to moderate the excitement; practise a salutary modesty that acknowledges the difficulty of personal change and of recovery. Perhaps rethink the metaphors of how mindfulness works — after all, picturing the exercise of present-moment awareness as mind-pumping that will make one more resilient to mental health bugs is probably not the most mindful of therapeutic models.
